# Rotavirus-NSP5 interacts with and hijacks host ATP citrate lyase to viroplasms, enhancing lipid biosynthesis for facilitating rotavirus infection

**DOI:** 10.1080/21505594.2026.2709258

**Published:** 2026-08-16

**Authors:** Ranjana Sharma, Shreya Banerjee, Ratul Datta Chaudhuri, Kaushik Kumar Dey, Animesh Gope, Shin-ichi Miyoshi, Daisuke Imamura, Mamta Chawla-Sarkar

**Affiliations:** a Regional VRDL, ICMR- National Institute for Research in Bacterial Infections (formerly ICMR-NICED), Kolkata, India; b Division of Applied Biosciences, ICMR- National Institute for Research in Bacterial Infections (Formerly ICMR-NICED), Kolkata, India; c Department of Central Instrumental Facility, ICMR- National Institute for Research in Bacterial Infections (Formerly ICMR-NICED), Kolkata, India; d ICMR- National Institute for Research in Bacterial Infections (Formerly ICMR-NICED), Collaborative Research Center of Okayama University for Infectious Diseases in India, Kolkata, India; e Graduate School of Medicine, Dentistry and Pharmaceutical Sciences, Okayama University, Okayama, Japan; f Research Center for Intestinal Health Science, Okayama University, Okayama, Japan

**Keywords:** Rotavirus, viroplasm, non-structural protein 5 (NSP5), proteomics, host-pathogen interaction, ATP citrate lyase

## Abstract

Rotavirus (RV) replication occurs within viroplasms (VMs) and is initiated by two RV non-structural proteins NSP2 and NSP5. Viruses exploit host cellular components for their replication and assembly; however, information on the roles of host proteins in VM dynamics and RV replication is limited. Thus, in the current study, we used proteomics to identify host proteins that interact with NSP5 during RV infection to delineate their role in virus replication. A large number of host proteins (*n* = 128) were found to interact with NSP5, and Gene Ontology enrichment analysis revealed the enrichment of various metabolic processes during RV infection. One of the essential host proteins, ATP citrate lyase (ACLY), which is involved in the *de novo* lipid synthesis pathway, was found to interact with C-terminal region of RV-NSP5 and colocalize within VMs. Following RV infection, the serine 455 phosphorylation of ACLY was induced, suggesting increased enzymatic activation. This correlates with enhanced lipid droplet production *via* increased acetyl-coenzyme A expression levels, which in turn supports VM formation. The ACLY inhibitors SB204990 or hydroxycitric acid tripotassium hydrate significantly reduced RV-infection *in vitro*. This anti-rotaviral effect of drug was further validated in BALB/*c* suckling mice by measuring viral protein expression and viral titers. In the presence of ACLY inhibitor, reduced viral titers, reduced viral protein expression, and improved small intestinal histopathology were observed. These findings suggest a protective effect of drugs against RV infection *in vivo* and highlight ACLY as a potential anti-rotaviral target for the development of new therapeutics.

## GRAPHICAL ABSTRACT

**Figure:**
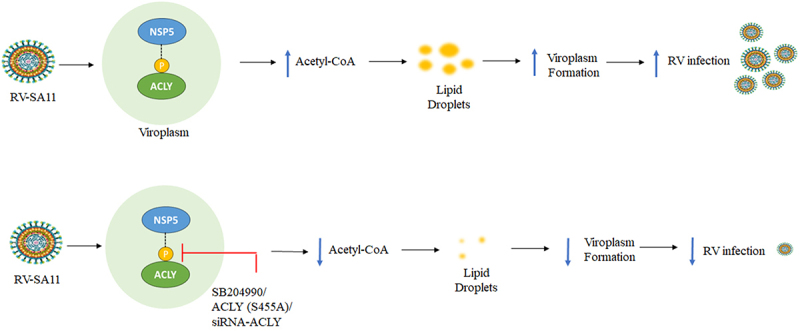
Rotavirus (RV)-NSP5 interacts with and co-opts ATP-citrate lyase (ACLY) within viroplasm (VM) for successful infection. RV-SA11 infection induces ACLY phosphorylation at ser455 which promotes acetyl-CoA and lipid droplet synthesis, driving VM formation and RV infection. Inhibition of ACLY phosphorylation by SB204990, ACLY S455A mutant, or ACLY-siRNA reduces acetyl-CoA production, thereby impairing VM formation and RV infection.

## Introduction

Rotaviruses (RVs) infect the small intestinal cells, leading to symptoms such as vomiting, diarrhea, and dehydration. For infants and young children, RV infections can be severe and life-threatening [1,2]. RVs are non-enveloped viral particles with a complex structure consisting of three concentric capsid layers surrounding an 11-segment double-stranded RNA (dsRNA) genome. This genome encodes six structural proteins (VP1-4, VP6, and VP7) that form the virus particles, and six non-structural proteins (NSP1-6, with NSP6 present in certain strains) involved in RV replication. The inner layer consists of viral RNA-dependent RNA polymerase (VP1), VP2, and the viral capping enzyme (VP3); the middle layer is composed of VP6; and the outer layer consists of VP7 and VP4 with the latter forming trimeric spikes. Viroplasms (VMs), which are essential for RV replication, are formed by both structural (VP6, VP1, VP2, and VP3) and non-structural RV proteins (NSP5, NSP4, and NSP2) [3,4]. Among these, NSP5 is a key player, as it can form viroplasm-like structures (VLSs) when co-transfected with either NSP2 or VP2 in an uninfected cell [5–8]. NSP5’s association with VMs is evident from the early stages of RV infection, that is, around 2 h post-infection (hpi). The number of VMs increases from 3 to 5 hpi, followed by their coalescence into larger VMs by 8 hpi [7,9,10]. During infection, NSP5 undergoes significant post-translational modifications such as O-linked glycosylation and phosphorylation at serine or threonine residues, which transforms the initially synthesized 26- to 28-kDa protein, into a larger 32- to 34-kDa form [11–14]. The interaction between NSP2 and NSP5 is crucial, as NSP2 binding triggers NSP5 phosphorylation [5,15]. The RV-encoded viroplasmic proteins NSP2 and NSP5 are subjected to liquid–liquid phase separation [16]. NSP5 has a high serine/threonine content, especially in its N-terminal region, followed by a highly charged basic–acid–basic (B–A–B) middle portion and a conserved C-terminus at the end [12,17]. Furthermore, the C-terminal region of NSP5 plays a key role in its hyperphosphorylation, multimerization, and interaction with NSP6, highlighting its multifaceted functions within VMs [18].

VMs are complex entities that comprise not only viral proteins and dsRNA but also a range of host components such as perilipin, which is associated with lipid droplets (LDs), components of the cytoskeleton such as tubulin, the host proteasome, and cellular chaperonins [19–24]. RV infection also causes the relocalization of host proteins to VMs, including several hnRNPs and ARE-BPs (known components of stress granules and P bodies), nuclear transport proteins (Exportin 1, Importin-β, Ran), cytoplasmic proteins (RPS8, VPS35, Sec31A) [25,26] and nuclear proteins (ATM and CHK2) [27].

VMs are closely associated with LDs, which are spherical structures with a neutral lipid core that contain triacylglycerol [28] and sterol esters. Neutral LD synthesis is a multi-step process regulated by different enzymes such as ATP citrate lyase (ACLY), acetyl-CoA carboxylase (ACC), fatty acid synthase (FASN), and diacylglycerol acyltransferase (DGAT) [29–32]. Fatty acids (FAs) provide essential building blocks for membrane biogenesis and signaling, and many positive‑strand RNA (+RNA) viruses such as Dengue virus (DV), Zika virus, and Hepatitis C virus (HCV) extensively exploit the host *de novo* fatty acid synthesis pathway to create the lipid environment needed for genome replication. By hijacking LD metabolism, viruses create locally enriched lipid environments that facilitate membrane remodeling, replication complex assembly, and energetic demands of genome replication [33]. This metabolic rewiring underscores the importance of lipogenic enzymes as critical host factors that can be exploited by viruses during infection.

During RV infection, various host proteins undergo relocalization and viroplasmic sequestration which may contribute to VM formation and maintenance. However, cells expressing only NSP5 cannot mimic the active infection scenario which is required to study the relocalization of host proteins [34]. Thus, to overcome this limitation, the current study was initiated to delineate the dynamic interaction of RV-NSP5 with host proteins during RV infection, thereby enabling subsequent analyses of the relocalization of host proteins into the VM. Co-immunoprecipitation with anti-NSP5 antibody in mock‑infected or RV-SA11‑infected cell extracts, followed by mass spectrometry, was performed to study the association of host factors with NSP5. This approach aimed to elucidate the complex network of host–virus interactions in VMs during infection to identify potential therapeutic targets. One of the NSP5-interacting partners was ACLY which is involved in lipid synthesis pathway.

ACLY is a large, single-polypeptide enzyme that functions as a homotetramer and is one of the central enzymes that links carbohydrate catabolism to lipid and cholesterol biosynthesis [35]. It catalyzes the conversion of mitochondrial-derived citrate and coenzyme A (CoA) into acetyl-CoA and oxaloacetate (OAA) by hydrolyzing ATP. Acetyl‑CoA is required for fatty acid and cholesterol biosynthesis, as well as for histone acetylation in the nucleus [36]. The expression of ACLY is transcriptionally regulated by sterol regulatory element binding protein 1 (SREBP1) [37]. The enzyme can be phosphorylated at multiple sites, including threonine 446 (Thr446), serine 450 (Ser450), and serine 454 (ser455-in humans and mice) [38,39]. Among them, phosphorylation at ser455 is the most widely studied and has been reported to enhance ACLY activity, promoting acetyl-CoA generation for lipid synthesis and histone acetylation [39–43]. AKT [40,44] and protein kinase A [38,40,45] are known to phosphorylate ACLY at ser455. However, some evidence suggests that ser455 phosphorylation may not always be essential for increased ACLY activity, highlighting possible context-dependent regulation [46].

Since lipids are critical for RV infection, previous studies have demonstrated that pharmacological disruption of fatty acid synthesis or LD homeostasis reduces VM formation [47–49]. Therefore, the functional relevance of ACLY was assessed *in vitro* through inhibitor-based studies, and the antiviral efficacy was further validated *in vivo* using BALB/*c* suckling mice model which physiologically mimics the disease observed in human infants, to assess antiviral efficacy.

## Materials and methods

### Cell culture and *in vitro* rotavirus infection

All experiments were performed using the African green monkey kidney cell line MA104 cells (ATCC number: CRL-2378^TM^). Cells were grown in minimal essential medium (MEM) supplemented with 10% fetal bovine serum (FBS; Gibco) and 1% antibiotic–antimycotic solution (Gibco) and were maintained at 37°C in 5% CO_2_ incubator. For viral infection, the cell culture-adapted rotavirus strain SA11 (G3P [2]) was primarily used to perform all experiments, whereas the human strain Wa was used for strain-independent analysis. For infection, the virus stock was diluted to a multiplicity of infection (MOI) of 3 and activated with 10 μg/ml acetylated trypsin (T6763, Sigma) at 37°C for 60 min in a water-bath. Next, the activated viruses were adsorbed onto MA104 cells and incubated for 1 h at 37°C in serum-free medium. Cells were washed with PBS (2 times) to remove any unbound virus, and infection was performed in fresh serum-free medium. All studies were performed at MOI of 3, and the time of virus removal after 1 h of adsorption was considered as 0-h post-infection (hpi). Drugs were added to the cells 1 h post-adsorption (0 hpi) for all the experiments [50] except for the pre-treatment (drug added 1 h prior to virus adsorption) and 5 hpi post-treatment (drug added at 5 h post-adsorption).

### Reagents and antibodies

SB204990 (HY-16450) and hydroxycitric acid tripotassium hydrate (HY-W009156) were purchased from MedChemExpress and dissolved in DMSO and water, respectively, to prepare 10 mM stock solutions. MEM (Gibco, 41500-067), FBS (Gibco, 10270-106), antibiotic–antimycotic (Gibco,15240063), trypsin with 0.05% EDTA (25300063), BODIPY 493/503 (D3922), rhodamine-conjugated goat anti-rabbit (35552), rhodamine-conjugated goat anti-mouse (35502), Dylight488-conjugated goat anti-rabbit (31670), Lipofectamine 2000 (11668019), mouse monoclonal antibody raised against ATP citrate lyase (MA517027) and c-MYC monoclonal antibody (13-2500) were purchased from ThermoFisher Scientific, USA. P-ACLY-S455 rabbit polyclonal antibody (AP0779) was purchased from Abclonal, USA. The protease inhibitor cocktail (P2714), phosphatase inhibitor cocktail 2 (P5726), MTT (M5655), siRNA universal negative control (si-NC; SIC001) and Acetyl-Coenzyme A assay kit (MAK039) were procured from Merck (Sigma-Aldrich, USA). Rabbit IgG antibody (3900S) was purchased from Cell Signaling Technology. Anti-β-actin HRP-conjugated (sc-47778) and mouse monoclonal antibodies against RV-VP6 (sc-101363), anti-FLAG (sc-166355) and normal mouse IgG antibody (sc-2025) were purchased from Santa Cruz Biotechnology (USA). The polyclonal rabbit antibodies against the RV non-structural protein, NSP5, NSP2 and structural protein VP6 were gifted by the Department of Virology and Parasitology, Fujita Health University School of Medicine, Aichi, Japan. The anti-VP6 antibody (rabbit) was specifically used in a single Co-IP experiment to validate nonspecific interactions with the IgG control. 5 nmol FlexiTube siRNA (pre-designed) against ACLY (1027417, GeneGlobe Id: SI02663339|S1) and 3’UTR siRNA-ACLY (1027416, GeneGlobe ID: GS47) were purchased from QIAGEN. 1436 pcDNA3 FLAG HA (10792), pEF6-ACLY (S455A) phosphomutant (70767), pEF6-ACLY (S455D) phosphomimetic (70768), pEF6-ACLY wild type (WT) (70765) were purchased from Addgene (USA). Protein marker (RM19001) was purchased from ABclonal.

### Rotavirus infection and treatment *in vivo*

The rotavirus strain SA11 was used for infection *in vivo* [50–53]. Four‑day‑old BALB/*c* suckling mice (*Mus musculus*) weighing nearly 3 g were collected from the animal house facility of ICMR-NIRBI, where mice were maintained in controlled pathogen-free conditions with the lactating dam and allowed access to water and standard chow diet *ad libitum*. Mice pups were inoculated with RV-SA11 of 10^6^ Plaque forming unit (PFU) through oral gavage on day 1. After 12 h of post-infection, mice were orally administered with either SB204990 (1 and 3 mg/kg/day) or an equal amount of DMSO vehicle through oral gavage for 3 days (from day 2 to day 4) at an interval of 24 h. Following euthanization by intraperitoneal administration of pentobarbital sodium at a dose of 100 mg/kg, the experimental mice were sacrificed on day 5. The small intestine of the animal was collected aseptically using sterile forceps and micro-scissor and the expression of viral protein (VP6) in the small intestinal homogenates of vehicle (positive control)- and drug-treated mice was quantified by Western blotting. For body weight measurement, mice were divided into 3 treatment groups including: (i) control/mock infected + treated with DMSO (negative control), (ii) RV-SA11 infected + treated with DMSO (positive control), and (iii) RV-SA11 + treated with SB204990 (1 mg/kg/day). The body weight of mice was measured daily from day 1 to day 7. Stool samples were collected from both infected (positive control) and drug-treated mice (on day 5), and a plaque assay was performed to quantify the RV particles present in the stool samples. At day 7, mice were sacrificed and small intestine, kidney and liver were collected. The organs were preserved in 4% (v/v) formalin for H&E staining. A subset of experiments was repeated for validation, using another drug, HCA (10 and 15 mg/kg/day) or water (as a vehicle). For body weight measurement, virus yield quantification and histology, 10 mg/kg/day of HCA was used. A total of 150 mice per drug were used for the study, which were allocated to different experimental groups by simple randomization. All experiments were performed in triplicate using *n* = 5 mice per group and a single animal was considered as an experimental unit. The sample size was determined based on previous reports [50,54]. Mice weighing <2 g were excluded to avoid variability in tolerance to infection and treatment. Control, infection and treatment groups were housed in separate cages under identical environmental conditions to prevent cross-exposure and were monitored daily. Group allocation was known only to the first investigator (RS), while the second investigator (RDC) who was blinded to the group identity, performed all the surgical procedures and data analysis. All experimental procedures in this study were adhered to the ARRIVE guidelines and Institutional Animal Ethical Committee.

### Ethical statement

The Institutional Animal Ethical Committee (IAEC), ICMR–National Institute for Research in Bacterial Infections (registration No. 68/GO/Rebi/S/1999/CPCSEA and project proposal no. PRO/2021/-May 2024-27) reviewed and approved the animal study.

### Quantitative real-time PCR (qRT-PCR)

Total RNA was extracted using TRIzol reagent (RNAIso Plus, Takara Bio, Cat No. 9109) following the manufacturer’s protocol from RV-SA11-infected mice intestinal tissue homogenate. To prepare cDNA, 1 μg of extracted RNA was reverse-transcribed according to the standard protocol [27]. Real-time PCR was performed in triplicate using PowerUp™ SYBR™ Green Master Mix (Applied Biosystems, Cat No. A25742) using QuantStudio 5. To quantify infection, primers used for real‑time PCR were specific to NSP4 (FP: 5’-GAAATGAGACGCCAGCTAGAA-3;‘ RP:5’-GTCGTTTGCACCGTCAATTTAT-3‘) and the housekeeping gene mouse-GAPDH (FP: 5’-GGTTGTCTCCTGCGACTTCA-3;‘ RP: 5’-TGGTCCAGGGTTTCTTACTCC-3’). The cycling conditions involved 40 cycles of 95°C for 15 s and 60°C for 1 min. Relative gene expression levels normalized to GAPDH were quantified using the formula 2− ^ΔΔCT^ (ΔΔCT = ΔCT_Sample_ − ΔCT_Control_), where CT is the threshold cycle.

### Transfection of siRNA and plasmid

siRNA and plasmid transfection in MA104 cells were performed using Lipofectamine 2000, according to the manufacturer’s instructions. After 30 h of transfection, the cells were infected with RV-SA11 and incubated for 5 h.

### Plasmid constructs and cloning

To study interaction-based experiments, NSP5 domains and full-length NSP5 were cloned into a FLAG-tagged pcDNA3 vector. For analysis of specific terminals or the middle region of NSP5 involved in interaction, the complete sequence of RV-NSP5 was divided into 3 non-overlapping domains containing (i) N-terminal: FLAG-F1 (residues 1–51), (ii) middle domain: FLAG-F2 (residues 52–150) and (iii) C-terminal: FLAG-F3 (residues 151–198). Specific primers were designed by incorporating Xho1 and Xba1 restriction sites into forward and reverse primers, respectively, for all domains (Table 1). These domains were amplified by PCR and cloned as XhoI/XbaI F1, F2, and F3-NSP5 domains into a pcDNA3 Flag HA-tagged vector (empty vector).

**Table 1.** List of NSP5 domain-specific primers. XhoI/XbaI restriction sites are mentioned within brackets.

Domains	Primers
FLAG-F1	F: 5’ TAAGCA(CTCGAG)GGATGTCTCTCAGTATTGACGTGGA 3’ (37 nts) R: 5’ TGCGGA(TCTAGA)CTAGTATTTATTGAATGCTTCTGCATCT 3’ (41 nts)
FLAG-F2	F: 5’TAAGCA(CTCGAG)GGATGCTGTCGAAGTCTCCAGAG 3’(34 nts) R: 5’ TGCGGA(TCTAGA)CTAAATTCTTGGGTAGTGCTTCCTA 3’ (40 nts)
FLAG-F3	F: 5’ TAAGCA(CTCGAG)GGGAAGCAGAGTCTGATTCAGAT 3’ (34 nts) R: 5’ TGCGGA(TCTAGA)CTATTACAAATCTTCAATCAATTGCATTGC 3’ (44nts)

### Co-immunoprecipitation (Co-IP)

Mass spectrometry study for NSP5-host interactome was performed in biological triplicates from both RV-SA11-infected (MOI 3) and mock-infected MA104 cells at 5 h post mock/RV-SA11 infection. Cells were lysed in IP lysis buffer containing 25 mM Tris, 150 mM NaCl, 1 mM EDTA, 1% NP-40, and 5% glycerol (pH 7.4). In the presence of a protease and phosphatase inhibitor cocktail, the cell lysates were pre-cleared for 1 h at 4°C using a control agarose resin slurry. A 5% aliquot of each cell lysate was used as an input control. Protein concentrations were determined using Bradford assay. Equal amounts of lysates were subjected to Co-IP with anti-NSP5 antibody using Pierce^TM^ Co-IP kit (26149, ThermoFisher Scientific) by following the manufacturer’s protocol. Briefly, specific antibodies were immobilized on AminoLink Plus coupling resin using sodium cyanoborohydride solution (5 M) on a rotator at room temperature for 2 h. The resin with immobilized antibody was washed six times using IP lysis/wash buffer and incubated with the pre-cleared cell lysates overnight at 4°C. After incubation, resin was washed six times using lysis buffer followed by 2 times with rinsing buffer (50 mM NH_4_HCO_3_ pH 8.0, 75 mM KCl, 2 mM EDTA). Rinsing buffer was used to remove trace amounts of NP-40 which may hinder with mass spectrometry. The lysates were eluted using elution buffer (0.5 M NH_4_OH pH 11.0–12.0, 0.5 mM EDTA) at 95°C for 10 min, and the eluted product was subjected to lyophilization [55]. The lyophilized product was sent to the mass spectrometry facility, National Center for Biological Sciences, Bangalore, India, for mass spectrometry analysis using MS/MS ion database search. A 1% global protein‑level false discovery rate (FDR) and significance threshold *p* < 0.006812 were applied for protein identification. Prior to mass spectrometry, the specificity of the pull-down was validated via Co-IP using anti-rabbit IgG control. Western blot analysis using anti-rabbit NSP5, anti-rabbit NSP2 and anti-rabbit VP6 confirmed the absence of nonspecific binding between IgG control and RV proteins (Figure S1).

For validation studies, Co-IP was performed using the Pierce^TM^ Co-IP kit as mentioned earlier, except for the last step where after incubation, the resin was washed using lysis buffer and immunoprecipitates were directly eluted by boiling at 95°C in elution buffer for 10 min and separated in SDS-PAGE followed by immunoblotting. Similarly, Co-IP was also performed using rabbit or normal mouse IgG antibodies in all experiments, which served as an isotype control. No interactions were detected in any IgG control samples (Figures S2A-D).

### Bioinformatic analysis

For bioinformatic analysis, two groups were selected: one with the proteins present in at least two different biological replicates of RV infection experimental sets and another group where proteins (those present in RV infection sets) were absent in control or mock‑infected MA104 cells. These groups were further sorted based on the presence of significantly unique peptide sequences. Proteins with more than one significantly unique peptide sequence were selected for analysis. The gene names (provided in mass spectrometry data; abbreviated as GN) for all proteins were uploaded into the STRING v12.0 (Search Tool for the Retrieval of Interacting Genes/Proteins) database to generate a protein–protein interaction network and gene ontology (GO) enrichments. Medium confidence (0.400) was used to generate the STRING network, and FDR ≤0.001 was used to generate GO enrichments.

### Treatment with chemical inhibitors

MA104 cells were treated with chemical inhibitors, SB204990 or HCA at 0 hpi and incubated for 5 h in most experiments. For the time-of-addition experiment, cells were treated with inhibitor either 1 h prior to infection (pre-treatment) or after virus adsorption at 0 hpi and 5 hpi (post-treatment). To assess viral protein expression, cell lysates were prepared and subjected to immunoblotting using antibodies specific for viral protein VP6.

### Immunoblot analysis

Cells were lysed using NP-40 cell lysis buffer supplemented with protease and phosphatase inhibitor cocktail. Protein concentration was estimated using Bradford assay (Sigma-Aldrich). The cell lysates were mixed with 4х Lammeli buffer and heated at 95°C for 10 min. The samples were separated by SDS-PAGE, transferred onto a PVDF membrane, blocked to reduce nonspecific binding, and probed with specific monoclonal or polyclonal antibodies. Primary antibodies were detected using HRP‑conjugated secondary antibodies (ThermoFisher Scientific^TM^). For all experiments, β-actin and VP6 antibodies were used as loading control and RV infection marker, respectively. Imaging was done using a chemiluminescent substrate (Millipore & Bio-Rad) within the ChemiDoc Imaging System (Bio-Rad) [56].

### Cell viability assay

MA104 cells, grown to 80%–90% confluency in 96-well plates, were treated with varying concentrations of either SB204990 (6.25 μM, 12.5 μM, 25 μM, 50 μM, and 100 μM) for 24 h or HCA for 72 h, and cell viability was measured by MTT assay. Untreated control cells were treated with respective vehicles for the drug (DMSO/water). For the assay, MTT solution dissolved in PBS at concentration of 5 mg/mL was added to the treated cells and incubated at 37°C for 4 h. The formazan complex was dissolved in DMSO and the optical density (OD was measured at 570 nm using a microplate reader (FLUOstar Omega, BMG Labtech). Cell viability percentage was determined by the formula (ODSample−ODBlank)×100/(ODControl−ODBlank). One hundered percent viability was considered for control cells (untreated) and viability of cells was represented as “percentage cell viability” [57].

### Plaque assay

Infectious virus particles were estimated using plaque assay. Briefly, MA1014 cells (70%–80% confluency) were seeded in 6-well dishes and infected with activated RV-SA11 using serial dilutions (10^2^- to 10^8^‑fold) for 1 h. Agarose overlay (0.7% agar in MEM supplemented with 1 μg acetylated trypsin/mL) was added to the cell monolayers after the media was removed and kept at 37°C for 2–3 days. Next, a second agar overlay (0.7% agar in MEM and 0.1% neutral red) was added and incubated at 37°C incubator until plaques were visible [50]. PFU was calculated as [58,59].

$$\begin{array}{c} \mathrm{PFU}/\mathrm{mL}=\mathrm{Number}\;\mathrm{of}\;\mathrm{plaques}/\left(\mathrm{dilution}\;\mathrm{factor}\;\;\right.\\ \;\;\;\;\;\;\;\;\;\;\;\;\times \;\mathrm{volume}\;\left.\mathrm{of}\;\mathrm{diluted}\;\mathrm{virus}\;\mathrm{added}\;\mathrm{to}\;\mathrm{theplates}\right) \end{array}$$

### Determination of IC_50_, CC_50,_ and SI

The 50% cytotoxic concentration (CC_50_) was determined using GraphPad Prism (Version 8) software by plotting the percentage cell viability against the corresponding drug concentrations. The 50% inhibitory concentration (IC_50_) of drugs was determined by plaque assay according to previously described protocol [50]. GraphPad Prism, non‑linear regression model: Y = Bottom+(Top−Bottom)/(1 + 10 (LogIC50−X) × Hill slope) was used to calculate both IC_50_ and CC_50_. The selectivity index (SI) was calculated by using the formula CC_50_/IC_50_ [60].

### Acetyl-CoA assay

Mock- or SA11-infected MA104 cells or siRNA-ACLY/inhibitor-treated cells were harvested, and the pellets were deproteinized and precipitated using perchloric acid (PCA). Briefly, samples were homogenized in 1 M PCA (1 mL/mg) by keeping the samples on ice, then centrifuged, and the supernatant was neutralized with 3 M potassium bicarbonate solution. The pH of the solution was verified (pH 6–8) and centrifuged to pellet potassium bicarbonate. Next, using these deproteinized samples, a reaction mix was prepared for each sample according to the manufacturer’s instructions. After 10 min of incubation in the dark at 37°C, fluorescence intensity (λex = 535/ λem = 587 nm) was measured using a microplate reader (FLUOstar Omega, BMG Labtech).

### Immunofluorescence

Immunofluorescence experiments were performed to detect the colocalization of RV-NSP5 and the cellular protein ACLY in the VM. Cells grown on coverslips were fixed for 10 min at room temperature using 4% (w/v) paraformaldehyde in PBS, permeabilized with 0.1% Triton X-100 for 20 min at 4°C, incubated with blocking solution (PBS supplemented with 2% (w/v) BSA) for 1 h at room temperature, followed by PBS wash and overnight incubation with NSP5 (raised in rabbit) and ACLY (raised in mouse) specific primary antibodies at 4°C. Unbound primary antibodies were washed with PBS followed by staining for 1 h at room temperature with DyLight 488-conjugated anti-rabbit and rhodamine-conjugated anti-mouse secondary antibodies. After washing, the slides were mounted with DAPI (4′,6′-diamidino-2-phenylindole) (Vector Laboratories, Burlingame, CA) and observed under a fluorescence microscope. For imaging, LSM 710 Axioplan microscope (Zeiss) (63×/1.4NA, oil immersion) was used. The acquired images were analyzed using Zen Blue software.

### BODIPY 493/503 staining for lipid droplet detection

For LD staining, MA104 cells were seeded on a coverslip placed in a 35 mm cell culture dish. At 60% confluency, cells were either infected with RV-SA11 directly or transfected with siRNA/plasmids followed by infection with RV-SA11. After the cells were fixed at room temperature for 30 min with 3% paraformaldehyde, they were incubated for 30 min with 1 μg/ml of BODIPY 493/503 (4,4-difluoro-1,3,5,7,8-pentamethyl-4-bora 3a,4a-diaza-s‑indacene; Thermo Fisher Scientific; 1-mg/ml stock solution was prepared in dimethyl sulfoxide; Molecular Probes). The coverslips were then washed 4 times with PBS, followed by overnight incubation in the dark with blocking buffer (BSA supplemented with 10% saponin) [61]. The next day, cells were washed, incubated with primary and secondary antibodies and mounted using DAPI as mentioned earlier. Excitation at 493 nm and emission at 503 nm were used for the imaging.

Cell fluorescence was measured using ImageJ, and Corrected Total Cell Fluorescence (CTCF) was calculated using the following formula:

$$\begin{array}{rl} & \mathrm{CTCF}=\mathrm{Integrated}\;\mathrm{Density}-\left(\mathrm{Area}\;\mathrm{of}\;\mathrm{selected}\right.\\ & \left.\;\;\;\;\;\;\;\;\;\;\;\;\;\;\;\;\;\mathrm{cell}\times \mathrm{Mean}\;\mathrm{fluore}\;\mathrm{scence}\;\mathrm{of}\;\mathrm{back}\;\mathrm{ground}\right) \end{array}$$

### Statistical analysis

Data analysis was based on the mean ± standard deviation (Mean ± SD) from at least three independent experiments (*n* ≥ 3). Student’s t-test (unpaired) was used for the acetyl-CoA experiment and for the comparison of CTCF and viral titers. Two-way ANOVA (Bonferroni’s multiple comparison test) was used for *in vivo* experiments to compare the effect of drug treatment and RV infection on body weight. For all performed experiments, *p* ≤ 0.05 was considered statistically significant. All statistical analyses were performed using GraphPad Prism (version 8.4.2).

## Results

### Host-protein ACLY interacts with RV-NSP5 and colocalizes within VM

Mass spectrometry analysis (LC-MS/MS) of NSP5 immunoprecipitates from mock‑infected and RV-SA11-infected MA104 cells identified 128-RV-NSP5-interacting proteins that were present in at least two infected biological replicates but absent in the control or mock‑infected sets (Table S1; Figure S3A). Further filtering of RV-NSP5-interacting proteins with more than one unique peptide sequence yielded 63 high-confidence proteins (Figure S3B). Gene Ontology (GO) enrichment analysis of these proteins revealed a diverse range of biological processes, molecular functions, cellular components, and pathways involved in RV-SA11 infection (Figure S3C-F). As biological processes revealed enrichment in primary metabolic pathways, host protein ACLY was selected for further investigation based on its probable functional relevance in the RV life cycle. ACLY is a pivotal enzyme in the *de novo* lipid synthesis pathway, generating acetyl-CoA required for lipid biosynthesis [29,62]. Since RV infection is known to rely on host lipid metabolism for efficient replication [19], we hypothesized that ACLY could be a potential host target involved in the RV life cycle.

**Figure 1.**
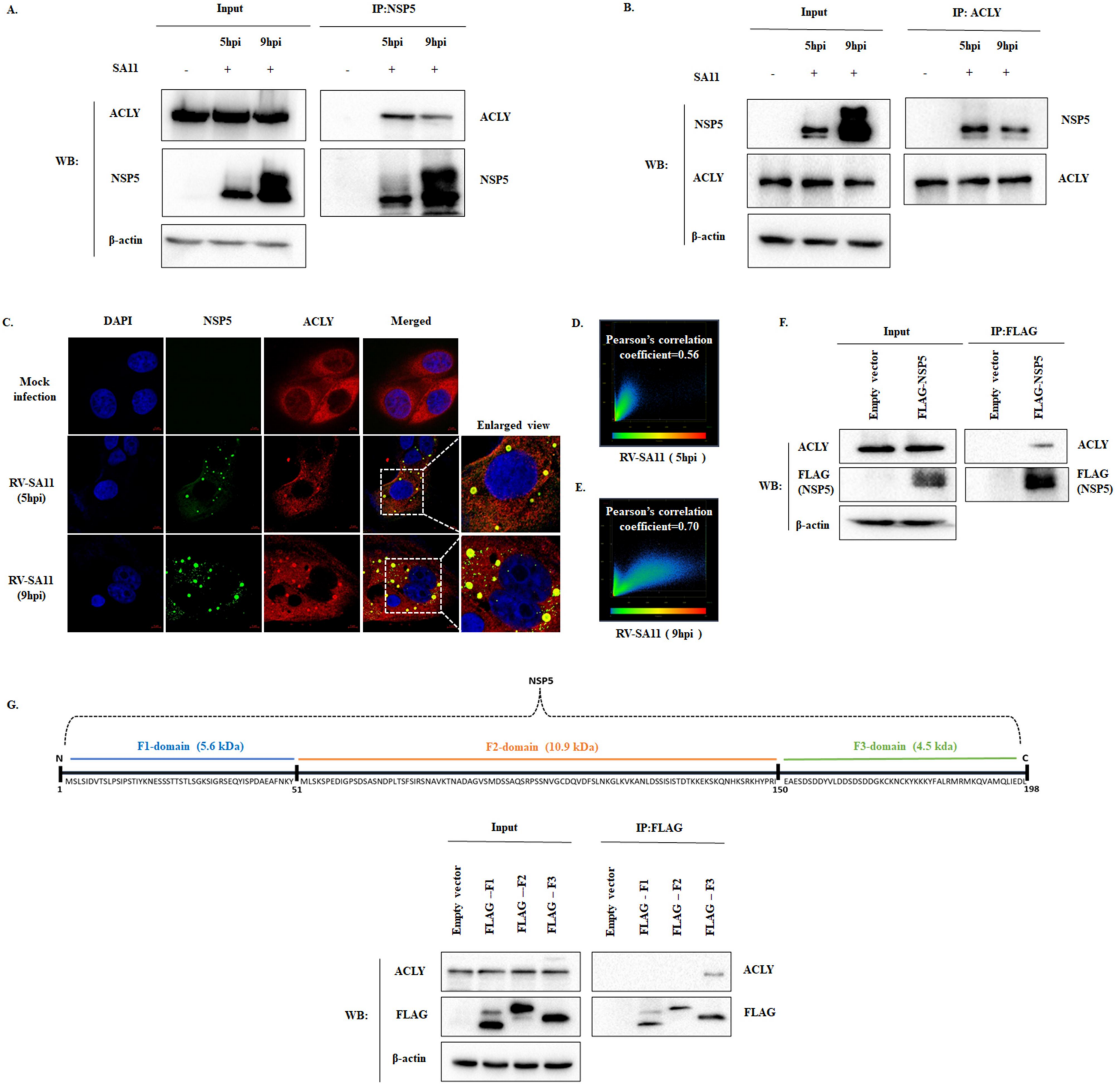
Host protein ACLY and RV-NSP5 interaction and viroplasmic colocalization. (A) Co-IP was performed in RV-SA11-infected MA104 cells at 5 hpi and 9 hpi using anti-NSP5 antibody followed by Western blot analysis with anti-ACLY antibody (right panel). (B) Reciprocal IP was performed using anti-ACLY antibody followed by Western blot analysis using anti-NSP5 antibody (right panel). (C) Immunofluorescence microscopy was performed to study colocalization of ACLY with viroplasmic protein NSP5. Both mock‑infected or RV-SA11-infected MA104 cells were fixed at 5 hpi and 9 hpi followed by primary staining with anti-NSP5 and anti-ACLY antibodies. Secondary staining was done using DyLight488-labelled anti-rabbit (for NSP5) and rhodamine-labelled anti-mouse (for ACLY) and mounting was done using 4′,6′-diamidino-2-phenylindole. For visualization of samples, LSM 710 Axioplan microscope (Zeiss) (63×/1.4NA, oil immersion) was used. Scale bar = 5 μm. Scattergrams of colocalized pixels of red and green channels of panel c were shown in (D) for 5 hpi and (E) for 9 hpi as obtained using Zen Blue software. Cell lysates were prepared from (F) empty vector- and FLAG-NSP5-transfected cells or from cells transfected with (G) FLAG-F1, FLAG-F2 and FLAG-F3 constructs and empty vector, followed by Co-IP using anti-FLAG antibody (for F and G; right panel). Inputs were probed with anti-ACLY, anti-NSP5 and β-actin antibodies (A and B; left panel) and with anti-ACLY, anti-FLAG and β-actin antibodies (F and G; left panel) for confirming protein expressions. β-actin was used as internal loading control for each immunoblot. Schematic representation of NSP5 domains is shown in G (upper panel). Successful pulldown was confirmed by immunoblotting with the same antibody used for IP (shown in each IP panel respectively).

To validate its interaction with RV-NSP5, Co-IP of mock‑infected and RV-SA11-infected lysates was performed using an anti-NSP5 antibody both at 5 and 9 hpi. It was observed that ACLY co-immunoprecipitated with NSP5 only in RV-SA11-infected cell lysates and not in control lysates, confirming the proteomic data (Figure 1(A)). Consistently, reciprocal-IP using anti-ACLY antibody also confirmed the interaction of ACLY with NSP5 (Figure 1(B)). The colocalization of ACLY within the VM in RV-SA11-infected MA104 cells at 5 and 9 hpi were assessed by immunofluorescence. Results suggest that ACLY colocalizes with RV-NSP5 within punctate VMs (Figure 1(C)), which is further supported by Pearson’s correlation coefficient values, i.e. 0.56 (Figure 1(D)) and 0.70 (Figure 1(E)), respectively. The Pearson’s coefficient was used to assess the degree of colocalization between ACLY and NSP5.

The mock-infected cells served as a negative control, where no such background signal was observed following incubation with NSP5 primary and anti-rabbit secondary antibodies, indicating absence of cross-reactivity of antibody with any host protein.

Although NSP5 is central to VM formation, mature VMs are formed by the involvement of multi-RV proteins, such as NSP2 and VP2. Hence, to determine whether the RV NSP5-ACLY interaction is direct, MA104 cells were transfected with either an empty vector or FLAG-NSP5 followed by Co-IP with anti-FLAG antibody. The data revealed that the interaction between ACLY and NSP5 was independent of other RV-encoded proteins (Figure 1(F)). Furthermore, to assess whether the terminal regions or the middle stretch of the NSP5 protein interacted with ACLY, the RV-NSP5 protein (198 aa) was divided into three domains viz. FLAG-F1 (1–51 aa), FLAG-F2 (52–150 aa), and FLAG-F3 (151–198 aa) (Figure 1G upper panel). MA104 cells were transfected with either an empty vector (pcDNA3 FLAG HA) or the plasmid encoding NSP5 domains, followed by Co-IP using anti-FLAG antibody. Result showed that ACLY interacted only with C-terminal of NSP5, i.e. F3 domain (Figure 1(G) lower panel). Collectively, these findings confirm that ACLY not only interacts with RV-NSP5 but also colocalizes with VMs, suggesting a crucial role of ACLY in the RV life cycle.

### RV-NSP5 induces ACLY ser455 phosphorylation to facilitate their interaction

**Figure 2.**
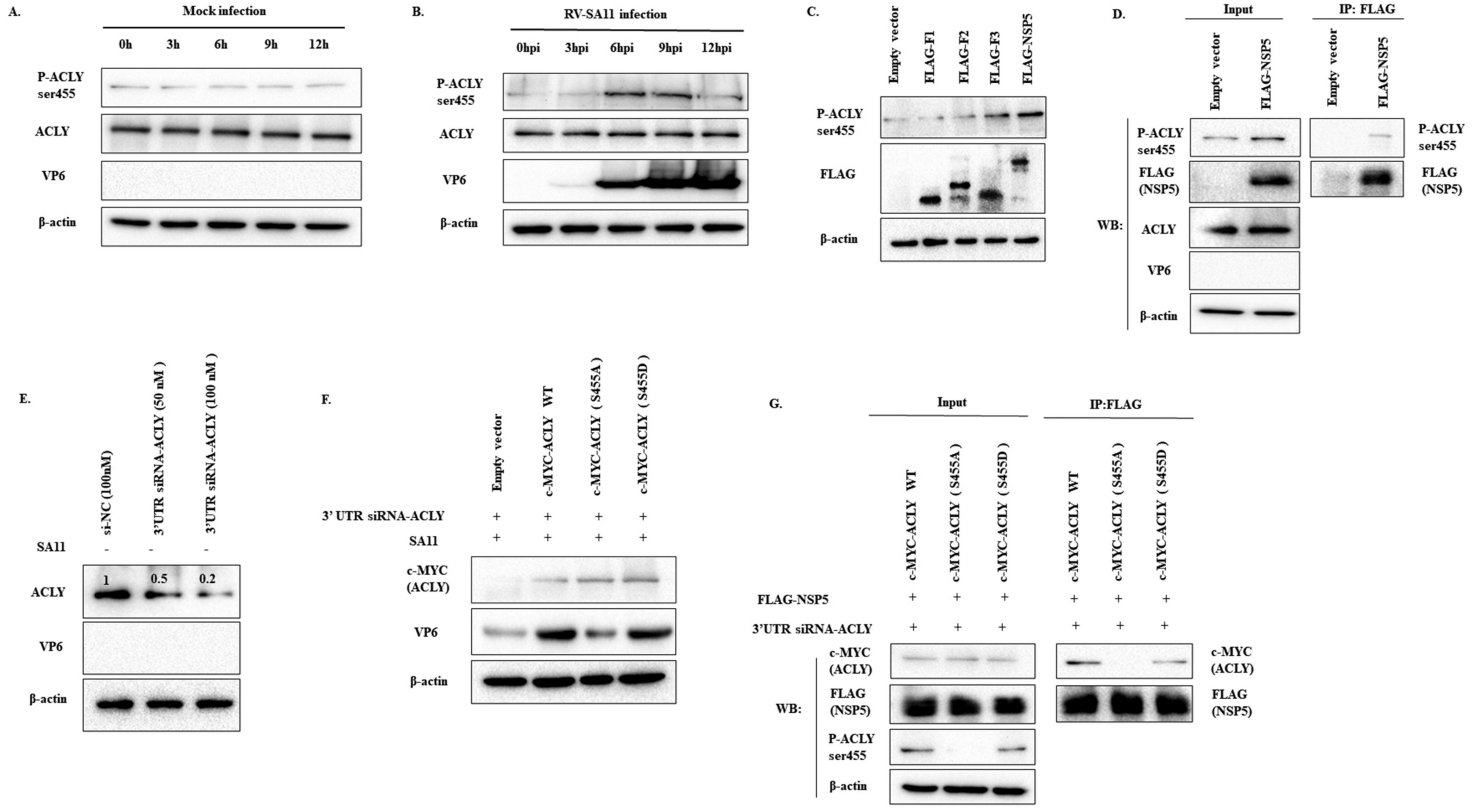
RV-NSP5 enhances P-ACLY ser455 which is crucial for RV-NSP5-ACLY interaction. At different time-points (0 h, 3 h, 6 h, 9 h, and12h), cell lysates were prepared from (A) mock‑infected and (B) RV-SA11-infected cells at MOI 3. For infected cells, the time of virus removal after 1 h of virus adsorption was considered as 0 h post-infection. Western blot analysis was done using anti-P-ACLY ser455, ACLY and VP6 antibodies for both A and B. (C) Different domains of NSP5 (F1, F2, and F3) and full‑length NSP5 were transfected individually to check the expression of P-ACLY ser455. Western blot analysis was done using anti-P-ACLY ser455 and anti-FLAG antibodies. (D) Co-IP was performed using anti-FLAG antibody from empty vector- and FLAG-NSP5-transfected cells, followed by Western blotting with anti-P-ACLY ser455 antibody (for IP panel). (E) Endogenous ACLY knockdown was confirmed using 3’UTR siRNA-ACLY (F) RV-SA11-infected endogenous ACLY knocked down MA104 cells were transfected with empty vector, c-MYC-tagged ACLY WT, ACLY (S455A), and ACLY(S455D) followed by immunoblotting using anti-MYC and anti-VP6 antibodies. (G) To knockdown endogenous ACLY, MA104 cells were treated with 3’UTR siRNA-ACLY followed by co-transfection of FLAG-NSP5 with c-MYC‑tagged ACLY WT, ACLY (S455A) and ACLY (S455D). Co-IP was performed using anti-FLAG antibody followed by Western blotting using anti-MYC and anti-FLAG antibody (right panel). Probing with the anti-FLAG antibody shown in IP panel confirmed the successful pulldown. Inputs were probed with anti-MYC, anti-FLAG and anti-P-ACLY ser455 antibodies (left panel). VP6 served as a RV-infection marker and β-actin served as a loading control.

The viroplasmic sequestration of ACLY and its interaction with NSP5 led us to analyze whether NSP5 had any impact on inducing ACLY phosphorylation during infection. Since in some cases, phosphorylation of ACLY, particularly at the ser455 residue, has been reported to enhance its enzymatic activity [40,41], we first investigated whether RV infection influences ACLY ser455 phosphorylation. To investigate whether RV infection had any impact on ACLY phosphorylation, immunoblotting was performed using cell lysate of RV-SA11-infected and mock‑infected MA104 cells at different time points (0, 3, 6, 9, and 12 h post‑mock/RV-SA11 infection). RV-SA11 infection revealed increased phosphorylation of ACLY at 6 and 9 hpi (Figure 2(B)) compared to the mock infection (Figure 2(A)). Consistent with previous observations (Figure 1(A,B)), the total ACLY level remained unaltered in both infected and mock‑infected cells (Figure 2(A,B)). To investigate whether the increase in ACLY phosphorylation is specific for only RV-SA11, a time-point experiment was performed in MA104 cells infected with other RV strains, that is, RV-Wa. Similar to the RV-SA11 strain, the RV-Wa strain also induced ACLY phosphorylation at 6 and 9 hpi compared to mock‑infected cells (Figure S4A,B). Hence, it can be concluded that the increase in ACLY phosphorylation during RV infection is not SA11 strain-specific. Additionally, following transfection of full-length FLAG-NSP5 or FLAG-F3 domain in the absence of infection, induced P-ACLY ser455 levels were observed as compared to the empty vector control, suggesting that RV-NSP5, more specifically its C-terminus, is the key driver of ACLY phosphorylation (Figure 2(C)). Moreover, P-ACLY ser455 also interacted with NSP5 in the absence of infection (Figure 2(D)). Next, the efficiency of 3’UTR siRNA-ACLY to knockdown the endogenous ACLY was confirmed in the mock-infected control cells. The result confirms the decrease in ACLY protein expression in 3’UTR siRNA-ACLY-treated cells (50 nM, 100 nM) compared to the negative control cells (si-NC, 100 nM) (Figure 2(E)). The importance of P-ACLY ser455 in RV infection was confirmed by assessing VP6 protein expression by Western blotting in endogenous ACLY knockdown (3’UTR siRNA-ACLY,100 nM treated) RV-SA11-infected cells transfected with c-MYC‑tagged ACLY WT or mutant ACLY (S455A or S455D). Reduced VP6 protein expression in ACLY (S455A) expressing cells compared to ACLY WT and restoration of VP6 protein expression in cells expressed with ACLY (S455D) directly demonstrates the crucial role of ACLY ser455 phosphorylation in positively regulating RV-infection (Figure 2(F)). c-MYC expression confirmed the successful transfection and expression of ACLY plasmids.

We next investigated whether ACLY phosphorylation is a pre-requisite for the interaction between NSP5 and ACLY. For this, endogenous ACLY was knocked down in MA104 cells using 3’UTR-ACLY-siRNA (100 nM) followed by co-transfection with either (i) FLAG-NSP5 and c-MYC-ACLY WT, (ii) FLAG-NSP5 and c-MYC-ACLY (S455A), or (iii) FLAG-NSP5 and c-MYC-ACLY (S455D). After Co-IP using an anti-FLAG (NSP5) antibody followed by immunoblotting with c-MYC antibody, the results showed that c-MYC (ACLY) interacted with FLAG (NSP5) in ACLY WT‑expressing cells. No interaction was observed between FLAG-NSP5 and the phosphomutant ACLY (S455A), whereas in the case of phosphomimetic ACLY (S455D), the interaction between c-MYC (ACLY) and FLAG (NSP5) was restored (Figure 2(G)). Taken together, these results confirmed that RV protein NSP5 induces phosphorylation of ACLY at ser455 residue which is required for ACLY-NSP5 interaction and successful infection.

### Inhibiting ACLY impairs RV infection *in* *vitro*

To assess the significance of ACLY during RV-infection and antiviral potency of ACLY inhibitors, RV-SA11 (MOI 3)-infected MA104 cells were treated with the ACLY inhibitor SB204990. It is a cell-penetrant γ-lactone prodrug of the active compound SB201076 that suppresses ACLY activity, thereby lowering cytosolic acetyl-CoA and reducing downstream *de novo* lipid synthesis [63].

**Figure 3.**
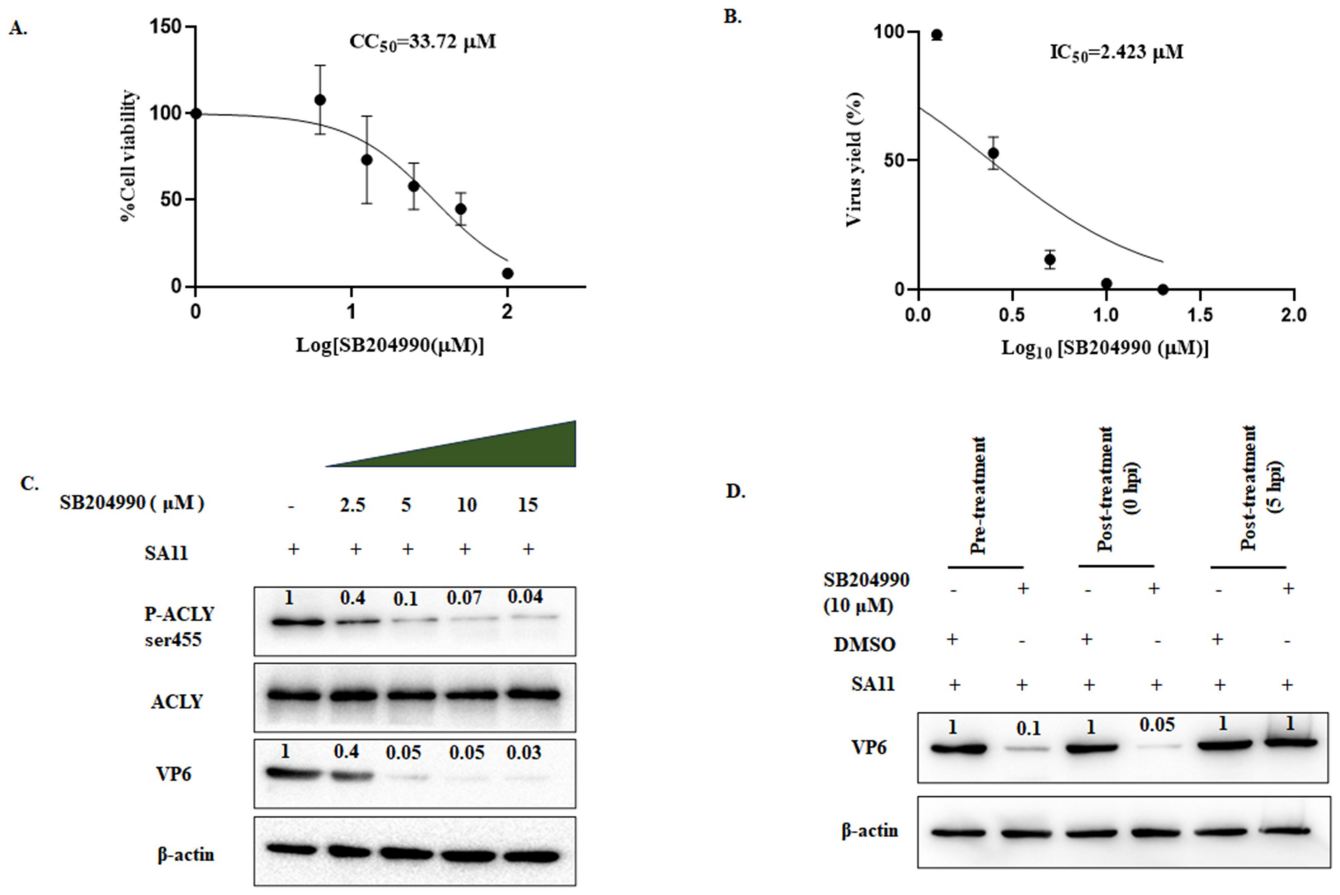
Inhibiting ACLY reduces RV infection *in vitro*. (A) CC_50_ of SB204990 in MA104 cells was assessed by MTT assay after 24 h of treatment with different doses of SB204990 (6.25 μM, 12.5 μM, 25 μM, 50 μM and 100 μM). (B) Increasing concentration of SB204990 (1.25 Μm, 2.5 μM, 5 μM, 10 μM and 20 μM) was administered to RV-SA11-infected MA104 cells at 0 hpi, and IC_50_ was calculated using plaque assay. (C) Western blot analysis was performed to study dose-dependent effect of SB204990 on RV-VP6 expression along with P-ACLY and total ACLY protein expression. (D) Time-of-addition experiment was performed to assess the stage of RV life cycle affected by SB204990. RV-SA11-infected MA104 cells were treated with DMSO or SB204990 (10 μM) either 1 h prior to infection (pre-treatment) or after 1 h of virus adsorption at 0 hpi and 5 hpi (post-treatment); RV-VP6 expression was analyzed by Western blotting after 9 h. β-actin was used as a loading control. For C and D, blots were normalized against β-actin. Each bar represents mean ± SD of three independent experiments.

Initially, the IC_50_ was determined by plaque assay by measuring infectious virus particles at increasing doses of SB204990 (2.5 µM, 5 µM, 10 µM, and 15 µM) added to the infected cells at 0 hpi. The treatment resulted in a dose-dependent reduction of viral titers with an IC_50_ of 2.423 μM (Figure 3(B)). This inhibitory effect occurred well below the cytotoxic concentration of drug (CC_50_ of 33.72 μM; Figure 3(A)), yielding an SI of 13.92, suggesting that the drug effectively suppresses viral infection at a nontoxic dose.

To further validate this finding, a dose–response study was conducted on RV-SA11‑infected MA104 cells, and the expression of the RV protein VP6 was assessed at 5 hpi by Western blotting. Consistent with the previous result, SB204990 (2.5 µM, 5 µM, 10 µM, and 15 µM) treatment at 0 hpi showed a steady and gradual dose-dependent depletion of viral protein VP6 and P-ACLY ser455 whereas total ACLY remained constant across all the concentrations (Figure 3(C)), suggesting that the inhibitor specifically targets ACLY phosphorylation. As SB204990 inhibits ACLY phosphorylation, loss of NSP5-ACLY interaction in FLAG-NSP5‑transfected cells treated with SB204990 further confirmed the importance of phosphorylation in interaction (Figure S4C). Time-of-addition experiments were performed to determine the stage at which SB204990 exerts its antiviral effect. Addition of SB204990 prior to virus adsorption (pre-treatment) or after adsorption of virus (0 hpi) significantly reduced VP6 protein expression compared to untreated controls. Post-treatment at 5 hpi had no significant effect on viral infection (Figure 3(D); S4D), suggesting that SB204990 acts at an early stage of the viral life cycle, prior to or at the immediate post-entry phase.

To determine any nonspecific effect of SB204990 on RV infection, ACLY was knocked down using siRNA (50 nM and 100 nM) in cells followed by RV-SA11 infection. Immunoblot analysis revealed a substantial reduction in RV-VP6 expression at 5 hpi compared to si-NC (negative control) (Figure S4E). Overall, the results suggest that SB204990‑mediated inhibition of ACLY phosphorylation results in reduced NSP5-ACLY interaction which is crucial for RV infection.

Similar experiments were performed using another ACLY inhibitor, hydroxycitric acid tripotassium hydrate (HCA), which is the major organic acid identified in *G. cambogia* fruit. It inhibits the enzymatic activity of ACLY which leads to a decrease in acetyl-CoA production [64–66]. The CC_50_, IC_50,_ and SI for HCA were determined to be >800 μM, 54.39 μM, and >14.71, respectively (Figure S5A,B). HCA treatment also reduced RV-VP6 expression; however, unlike SB204990, it reduces total ACLY levels in a dose-dependent manner (Figure S5C).

### Inhibiting ACLY impairs RV infection *in* *vivo*

**Figure 4.**
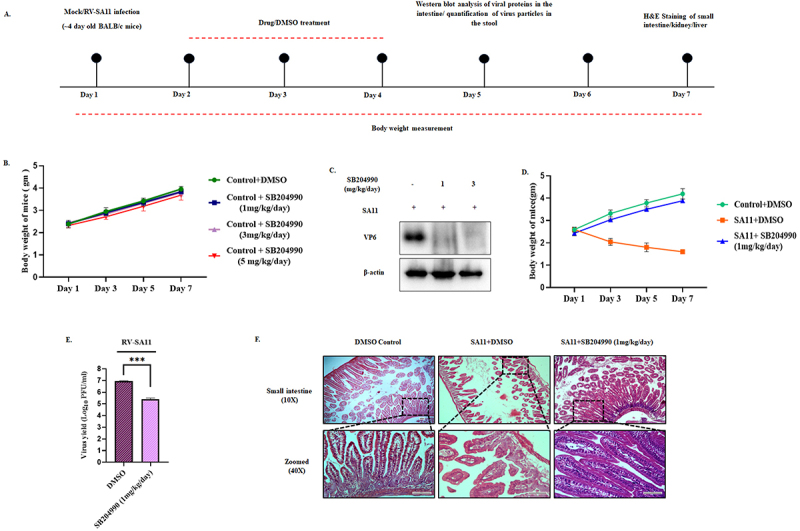
Inhibiting ACLY reduces RV infection *in vivo* (BALB/*c* suckling mice). (A) Workflow of RV-SA11 infection and SB204990 treatment in mice. (B) To assess cytotoxicity of SB204990, non-infected suckling mice were orally treated with drug at 1 mg/kg/day, 3 mg/kg/day, and 5 mg/kg/day (from day 2 to day 4) and body weight of mice was measured from day 1 to day 7. (C) RV-SA11-infected mice were treated with either DMSO vehicle or SB204990. On the first day, suckling mice were infected with RV-SA11, and after 24 h of infection, the treatment group was administered with SB204990 (1 mg/kg/day and 3 mg/kg/day) for 3 days. Intestinal homogenates were prepared after 3 days, and the expression of VP6 protein (infection marker) and β-actin (loading control) was analyzed by Western blotting using anti-VP6 and anti- β-actin antibodies. (D) Mice were divided into mock-infected, RV-SA11 infected, and RV-SA11 infected + SB204990 (1 mg/kg/day) treatment groups. Body weight was monitored throughout the experiment to evaluate recovery from RV-induced weight loss following SB204990 treatment. (E) Stool samples from infected and SB204990 (1 mg/kg/day)-treated mice were collected, and virus yield was quantified from the stool samples at day 5 using plaque assay. (F) Morphology of the small intestinal tissue was studied by H&E staining of mock‑infected, RV-SA11-infected and SB204990‑treated mice. Scale bars = 50 μm. Two-way ANOVA followed by Bonferroni’s multiple comparisons test was used to compare the cytotoxicity of SB204990 at different doses (*p* = 0.320) and for comparing body weight of RV-SA11-infected mice with SB204990 treatment (*p* ≤ 0.001) or control mice (B and D, respectively). Each bar represents mean body weight of mice ±SD of three independent experiments. Unpaired Student’s t-test (for comparing the results of viral titer from stool samples) was used to determine the level of significance (***p ≤ 0.001). For each experiment, *n* = 5 mice were used.

Next, the positive role of ACLY in RV pathogenesis was further validated using an animal model. To assess the cytotoxicity or growth-inhibitory effects of SB204990, the body weight of BALB/*c* suckling mice was monitored daily after vehicle or SB204990 (1, 3, and 5 mg/kg/day) treatment for 3 days (Figure 4(A)). Treatment with SB204990 at different doses did not show any significant change in the body weight of mice (Figure 4(B)). Next, to investigate the anti-rotaviral activity of SB204990, mice were orally administered with RV-SA11 on day 1. After 24 h post-virus inoculation, SB204990 1 mg/kg/day and 3 mg/kg/day or DMSO vehicle was orally administered to the mice for three consecutive days (day 2–4). Mice were sacrificed 24 h after the last treatment (on day 5) and the expression of RV-VP6 from small intestinal homogenates was assessed. Immunoblotting result showed a significant reduction in RV-VP6 expression in RV-SA11‑infected mice treated with SB204990 at both 1 mg/kg/day and 3 mg/kg/day compared to DMSO-treated infected mice, suggesting the anti-rotaviral activity of SB204990 (Figure 4(C)). As SB204990 at 1 mg/kg/day showed antiviral activity, we proceeded with 1 mg/kg/day dose for the rest of the experiments. Following SB204990 treatment, the mice also showed recovery in body weight loss due to RV infection (Figure 4(D)). Infectious RV particles in the stool samples of RV-SA11-infected mice treated with either DMSO or SB204990 were quantified (on day 5). The data revealed a significant reduction in RV particles in the stool samples of SB204990‑treated mice compared with that in untreated mice (Figure 4(E)). Consistent with this data, SB204990 treatment in RV-infected mice restored intestinal villus morphology that was damaged due to infection (Figure 4(F)). H&E staining of kidney and liver tissue samples from non-infected control (vehicle-treated) and SB204990-treated mice also confirmed the non-cytotoxic effect of 1 mg/kg/day dose of SB204990 (data not shown).

Similarly, to evaluate the cytotoxic effect of HCA, mice were treated with 5, 10, and 15 mg/kg/day of HCA, and body weight was monitored. Even at a higher dose of 15 mg/kg/day, HCA did not show any significant reduction in body weight compared to untreated control mice (Figure S5D). Treatment of RV-SA11-infected mice with HCA at dose 10 or 15 mg/kg/day resulted in reduced expression of the viral protein VP6 (Figure S5E). As 10 mg/kg/day HCA sufficiently reduced viral infection, this dose was administered in all experiments. RV-infection-induced body weight loss in mice was recovered and virus yield from stools was significantly reduced after HCA treatment (Figure S5F,G). Intestinal morphology was also restored in mice treated with HCA (Figure S5H), and the tissue morphology of the kidney and liver was not compromised (data not shown).

Collectively, these results validate that pharmacological inhibition of ACLY by SB204990 or HCA effectively suppresses RV infection *in vivo*, without causing significant toxicity and highlights ACLY as a potential antiviral therapeutic target against RV infection.

### NSP5-induced ACLY phosphorylation is associated with enhanced acetyl-CoA production and LD formation during RV infection

Previous results revealed a positive role of P-ACLY in RV infection; thus, we next investigated whether NSP5-mediated ACLY ser455 phosphorylation contributes to acetyl-CoA production. ACLY catalyzes the conversion of citrate into cytosolic acetyl-CoA; therefore, enhanced ACLY enzymatic activity is expected to promote acetyl-CoA production and consequently support downstream *de novo* lipid synthesis [40,62,67,68].

**Figure 5.**
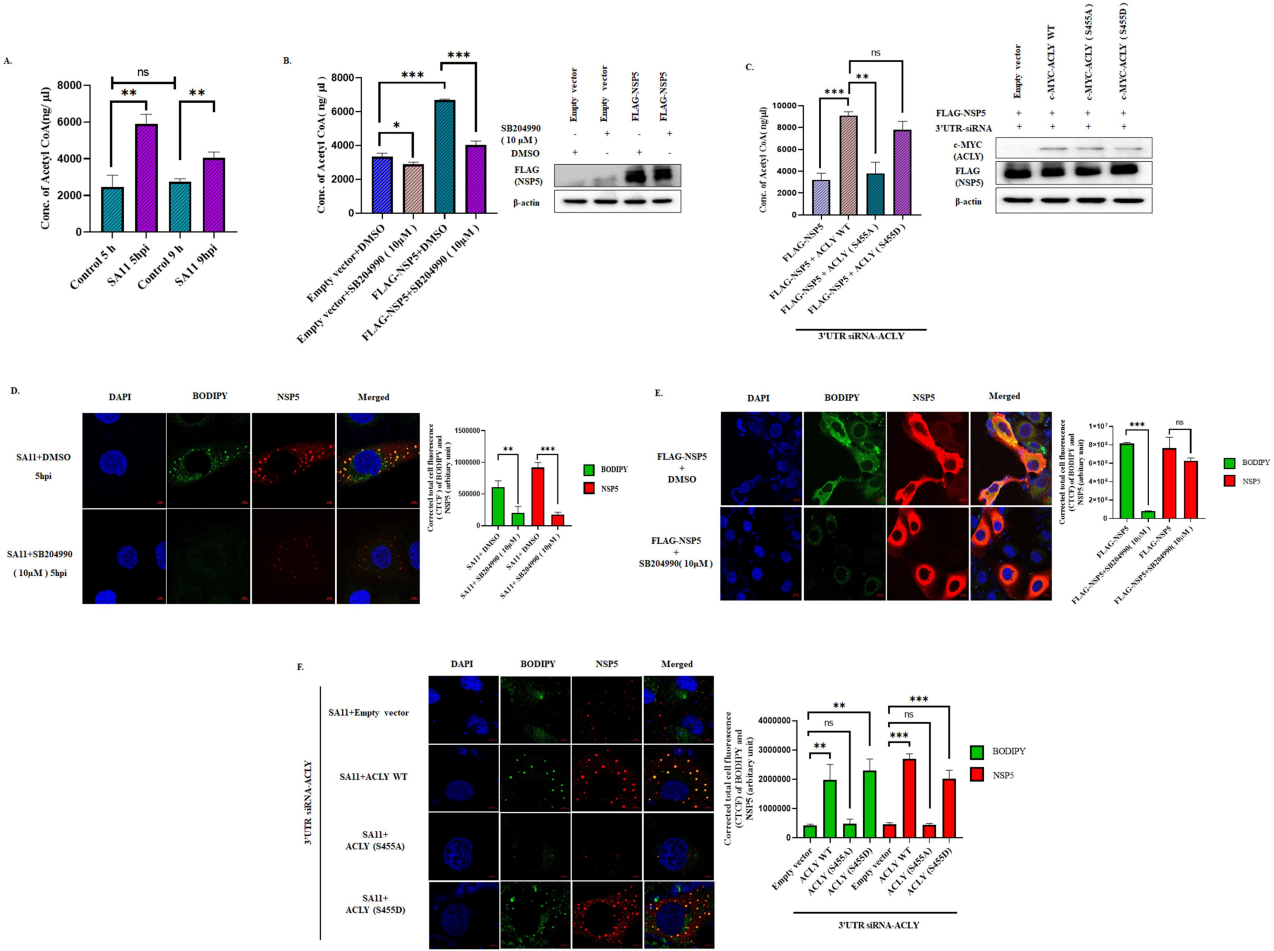
P-ACLY ser455 induced by RV-NSP5 for production of acetyl-CoA is crucial for LD synthesis and VM formation. Cell pellets were deproteinized and processed for acetyl-CoA assay from (A) mock‑infected or RV-SA11-infected cells (5 hpi and 9 hpi), (B) control empty vector- and FLAG-NSP5-transfected cells treated either with DMSO or SB204990 (10 μM) (left panel); FLAG-NSP5 transfection was confirmed by immunoblotting using anti-FLAG antibody (right panel). (C) Result of acetyl-CoA assay from endogenously ACLY knocked down cells co-transfected with FLAG-NSP5 and c-MYC‑tagged ACLY WT, ACLY (S455A) and ACLY (S455D) (left panel); both FLAG-NSP5 and c-MYC-ACLY plasmid expression was confirmed by immunoblotting (right panel) using anti-FLAG and anti-c-MYC antibodies respectively. Immunofluorescence imaging was done to visualize VMs and LDs. VMs were detected using anti-NSP5 antibody followed by rhodamine-conjugated secondary antibody (red), while LDs were stained with BODIPY 493/503 (green) in cells with (D) RV-SA11 infection (5 hpi), treated either with DMSO or SB204990 (10 μM) (E) FLAG-NSP5-expressing cells treated either with vehicle or SB204990 (F) endogenously ACLY knocked down cells transfected with c-MYC‑tagged ACLY WT or ACLY (S455A) or ACLY (S455D), followed by RV-SA11 infection. CTCF for BODIPY 493/503 and NSP5 is provided in the right panel for D, E, and F. Scale bar 5 μm was used. Unpaired t-test (for acetyl-CoA assay and for comparing CTCF of BODIPY 493/503 and NSP5) was used to determine the level of significance (ns denotes *p* value not significant, *p ≤ 0.031, **p ≤ 0.001, ***p ≤ 0.001). Each bar is a representation of mean ± SD of three independent experiments.

To determine the impact of RV infection on acetyl-CoA levels, MA104 cells were infected with SA11, and acetyl-CoA assay was performed at 5 hpi and 9 hpi. RV infection resulted in a significant increase in acetyl-CoA concentration (*p* ≤ 0.05) at both 5 and 9 hpi compared to that in the controls (Figure 5(A)). Furthermore, it was observed that acetyl-CoA levels were also elevated in FLAG-NSP5-transfected cells (*p* ≤ 0.001), whereas ACLY knockdown significantly reduced acetyl-CoA levels in FLAG-NSP5‑expressing cells relative to si-NC, negative control,100 nM (*p* ≤ 0.001) (Figure S6A).

We next evaluated whether phosphorylation of ACLY contributes to acetyl-CoA production. To assess this, NSP5-transfected cells were treated with the ACLY inhibitor SB204990 (10 µM), which specifically inhibits ACLY phosphorylation without affecting the total ACLY levels. FLAG-NSP5 expression was first confirmed by immunoblotting (Figure 5(B), right panel), following which acetyl-CoA levels were measured. Acetyl-CoA assay revealed that SB204990 treatment significantly reduced acetyl-CoA levels in FLAG-NSP5‑transfected cells compared to untreated controls (*p* ≤ 0.001) (Figure 5(B), left panel).

Furthermore, to specifically assess the importance of phosphorylation at ser 455 residue of ACLY, endogenous ACLY was knocked down using 3’UTR siRNA-ACLY (100 nM), followed by co-transfection of FLAG-NSP5 with c-MYC-tagged-ACLY WT, ACLY (S455A), or ACLY (S455D). Following confirmation of c-MYC-tagged-ACLY plasmids expression (Figure 5(C), right panel), acetyl-CoA levels were quantified. Acetyl-CoA production was found to be increased in cells expressing ACLY WT, but reduced acetyl-CoA production was observed in ACLY (S455A)-transfected cells, whereas levels were restored in phosphomimetic ACLY (S455D)-expressing cells (Figure 5(C), left panel). Together, the results suggest that NSP5 expression alone is sufficient to induce acetyl-CoA production through ACLY ser455 phosphorylation.

Furthermore, to understand whether the reduction in acetyl-CoA levels upon ACLY inhibition or knockdown has any impact on LD or VM formation, RV-infected (5 hpi) MA104 cells were either treated with SB204990 (10 µM) at 0 hpi, or siRNA-ACLY (100 nM). LDs were visualized using BODIPY 493/503 staining, whereas NSP5 was stained with rhodamine. Immunofluorescence imaging revealed a significant decrease in CTCF of BODIPY and rhodamine (NSP5) in cells treated with SB204990 (Figure 5(D)) or ACLY-siRNA (Figure S6B), compared to the vehicle‑treated infected cells, suggesting reduced LD and VM formation. Moreover, a significant reduction in the CTCF of BODIPY and rhodamine (NSP5) was observed in SB204990 (10 µM)-treated-FLAG-NSP5-transfected cells compared to DMSO control (Figure 5(E)). To rule out whether the reduction in LDs was a direct consequence of ACLY inhibition or an indirect effect due to decreased RV infection, uninfected control cells were treated with either SB204990 or DMSO vehicle. A significant reduction (*p* ≤ 0.001) in LDs after SB204990 treatment was observed in uninfected control cells (Figure S6C), confirming a direct effect of ACLY inhibition on lipid synthesis. Consequently, the observed decrease in VM formation can be attributed to the suppression of ACLY-dependent LD synthesis. Consistently, immunofluorescence imaging revealed reduced LD synthesis and VM formation in ACLY (S455A)‑expressing cells compared to ACLY WT- or ACLY (S455D)-transfected cells (Figure 5(F)), confirming essential role of P-ACLY ser455 in lipid synthesis and VM formation.

Collectively, these findings highlight the crucial role of NSP5-induced ACLY ser455 phosphorylation-mediated LD synthesis which supports VM formation during RV infection.

## Discussion

The present study establishes an unexplored role of ACLY in modulating RV pathogenesis by enhancing acetyl-CoA production and LD formation during VM assembly. Although the involvement of ACLY in viral infections has so far been limited to a few studies on HBV, SARS-CoV-2 and VACV [69–71], this is the first report which identifies a viral protein, RV-NSP5 to be physically associated with the host ACLY protein, thereby inducing its phosphorylation-dependent activation. This study provides a comprehensive and realistic snapshot of the host proteins that associate with the viroplasm-nucleating protein NSP5 physically in a manner stable enough to influence infection dynamics, and in a definite infection scenario. To this end, we used RV-infected cells to analyze the NSP5 interactome by mass spectrometry following co-immunoprecipitation with anti-NSP5. Extending from recent reports that either mimic infection by NSP5 plasmid transfection or utilize advanced proximity interactome techniques for detecting neighboring proteins that may or may not physically interact [24,26], our data revealed many novel host proteins in the NSP5 interactome (https://doi.org/10.6084/m9.figshare.29148377.v2). Notably, our mass spectrometry study also identified several known NSP5-interacting proteins (denoted by an asterisk (*); S1 Table) [24,26] further validating the data.

In congruence with the presently validated NSP5-ACLY interaction, ACLY was found to colocalize within VMs, indicating its possible role in the viral replication. Notably, both RV-SA11 infection and exogenous expression of full-length NSP5 alone could induce ACLY ser455 phosphorylation and corresponding escalation in acetyl-CoA production, suggesting that NSP5 mediates these effects independent of any other viral protein.

The NSP5 C-terminal region (151–198 residues) was sufficient for binding and inducing ACLY phosphorylation. The last ten C-terminal amino acids (189–198) of NSP5 have been previously shown to bind with viral proteins such as NSP6 [18] and VP2 [72], as well as for oligomerization of NSP5 into decamers [18,73]. The precise NSP5 residue(s) inducing ACLY activation and their role in viral replication remain to be determined.

Cells expressing ACLY (S455A) phosphomutant significantly reduced NSP5-ACLY interaction, acetyl-CoA levels, LD synthesis, and VM formation resulting in reduced RV infection. In contrast, ACLY (S455D) phosphomimetic promoted stable NSP5-ACLY interaction and restored RV infection. P-ACLY levels peaked during the early hours of infection (6 hpi and 9 hpi) which correlated with NSP5-ACLY interaction and increased acetyl-CoA levels. This metabolic spike aligns with the critical demands for LDs during the early hour of infection to serve as platforms for VM organization and viral RNA replication [47]. At later stages of infection, when VMs are established, the demand for *de novo* lipid synthesis may diminish, or once sufficient lipid pools are generated for viral replication, the ACLY phosphorylation may be deactivated as a feedback mechanism.

Furthermore, treatment with two potent inhibitors of ACLY, SB204990 and HCA, known to competitively bind to the citrate-binding site of ACLY, resulted in reduced RV-SA11 infection *in vitro* in a dose-dependent manner. While HCA downregulated both total ACLY and P-ACLY levels, SB204990 specifically targeted ACLY phosphorylation, which inhibits NSP5-ACLY interaction and acetyl-CoA production, further confirming the functional importance of ACLY phosphorylation during infection. The time-of-addition experiment confirmed the importance of ACLY during the early stage of the RV life cycle, as SB204990 retained antiviral activity when administered prior to infection or immediately after infection but was ineffective when added at later time points. Thus, RV exploits NSP5 to co-opt host lipid biosynthetic machinery through phosphorylation-dependent activation of ACLY at ser455, rendering this axis indispensable for productive infection.

The *in vitro* anti-rotaviral effect of ACLY inhibitors (SB204990 and HCA) was also validated in an animal model (BALB/*c* suckling mice). Drugs were administered to mice for three consecutive days because SB204990 levels have been reported to decrease significantly after 24 hrs of treatment in young mice [74], whereas RV-SA11 infection and disease severity in suckling mice peaked within 3–4  days after 24 h of post-inoculation [52]. Treatment with either compound led to a marked decrease in viral protein VP6 expression within the small intestine and a significant reduction in infectious virus particles in the stool. A notable recovery from RV infection-induced symptoms such as restoration of body weight loss and healing of damaged intestinal villi was also observed in mice treated with ACLY inhibitors, suggesting a strong anti-rotaviral effect *in vivo*.

The findings in BALB/*c* neonates are likely to be generalized to other mammals that are known to be infected by RV such as infants of humans, ruminants, porcine, and equine animals that show similar symptoms upon infection. However, as the antiviral effect of drugs was studied in a limited number of animals under controlled laboratory conditions, therefore, further preclinical investigations including larger animal studies and human trials are required to fully assess the anti-rotaviral efficacy of ACLY inhibitors.

Keeping in view that the present report is the first to explore the functional aspect of the NSP5-ACLY interaction, we focused on establishing the role of ACLY in modulating RV pathogenesis. Though RV infection or full‑length NSP5 or NSP5-F3 domain overexpression potentiated the enzymatic activity of ACLY through phosphorylation, direct demonstration that NSP5-mediated ACLY activation is solely responsible for enhancing virus replication remains unanswered as NSP5 is indispensable for VM formation and RV replication [75–77]. Thus, silencing NSP5 or loss of function study was not feasible. However, SB204990, a drug which specifically inhibits ACLY phosphorylation without altering total ACLY expression, exhibited reduced VM formation and RV replication. Furthermore, in cells where endogenous ACLY was suppressed using 3’UTR siRNA and ACLY phosphomutant was expressed, significant reduction in SA11 infection was noted. Thus, collectively it can be hypothesized that NSP5-mediated ACLY phosphorylation positively regulated RV infection.

In conclusion, this study provides valuable insights into the dynamics of ACLY and RV-NSP5 during RV replication. Therefore, targeting ACLY may offer a promising therapeutic strategy for combating RV infection. As lipid biosynthesis plays a role in the biogenesis of many viruses, future studies may explore the role of ACLY in other viruses to develop pan-antiviral therapeutics.

## Supplementary Material

S 6 ACLY knockdown reduces acetyl CoA production and LDs formation.jpg

Supplementary figure legend clean version.docx

S 3 RV NSP5 host protein interactome.jpg

S1 Table List of NSP5 interacting host proteins from mass spectrometry data which are only present in RV SA11 infection sets at 5hpi.xls

S 5 ACLY inhibitor HCA shows anti rotaviral activity both in vitro and in vivo.jpg

S 2 Absence of non specific interactions in Co IP with IgG antibody.jpg

Supplementary table legend clean version.docx

S 4 RV Wa infection induces ACLY ser455 phosphorylation ACLY phosphorylation promotes NSP5 ACLY interaction its knockdown impairs RV infection and SB204990 inhibits infection.jpg

S 1 Anti rabbit IgG antibody does not cross react with RV proteins.jpg

## Data Availability

Metadata of proteomics analysis using anti-NSP5 antibody for Co-IP from mock- and RV-SA11-infected MA104 cells at 5 hpi has been deposited to FigShare and is openly available at DOI: https://doi.org/10.6084/m9.figshare.29148377.v2 [78] under a CC BY 4.0 license. All the raw dataset supporting the findings of this study along with the supplementary materials have been deposited to FigShare and are openly accessible at DOI: https://doi.org/10.6084/m9.figshare.31444165 under a CC BY 4.0 license [79].
