# Supplementary material for: Rotavirus-NSP5 interacts with and hijacks host ATP citrate lyase to viroplasms, enhancing lipid biosynthesis for facilitating rotavirus infection
Source: Virulence. 2026 Aug 16;17(1):2709258. doi: 10.1080/21505594.2026.2709258 (PMC13483473; doi:10.1080/21505594.2026.2709258)
Supplement: Supplementary file 1 [file KVIR_A_2709258_SM5211.docx]

**Supplementary table legend:**

**S1 Table:** **List of NSP5-interacting host proteins from mass spectrometry data that are only present in RV-SA11 infection sets at 5hpi.** Proteomic analysis was performed using three biological replicates (A1, A2, A3 and B1, B2, B3). Where samples A and B serve as technical replicates. The data here represents the combined scores for A and B calculated across their respective three biological replicates. *Proteins denoted by an asterisk (*) have been previously identified as NSP5-partners in independent studies. Average (emPAI) represents the mean exponentially modified Protein Abundance Index across three replicates. Log(e) and Log(I) indicate the natural log transformation of emPAI and protein intensity values, respectively.
