# Supplementary material for: Rotavirus-NSP5 interacts with and hijacks host ATP citrate lyase to viroplasms, enhancing lipid biosynthesis for facilitating rotavirus infection
Source: Virulence. 2026 Aug 16;17(1):2709258. doi: 10.1080/21505594.2026.2709258 (PMC13483473; doi:10.1080/21505594.2026.2709258)
Supplement: Supplementary file 2 [file KVIR_A_2709258_SM5216.docx]

# **Supplementary figure legend:**

**Fig S1: Anti-rabbit IgG antibody does not cross-react with RV proteins.** Co-IP using an anti-rabbit IgG isotype control and probing with RV non-structural and structural antibodies (anti-NSP5, NSP2 and VP6 antibody) for western blotting confirms the absence of non-specific interaction.

**Fig S2: Absence of non-specific interactions in Co-IP with IgG antibody.** (**A**) Co-IP was performed in RV-SA11 infected MA104 cells at 5hpi and 9hpi using rabbit IgG antibody followed by immunoblotting with anti-ACLY antibody (IP panel). (**B-D**) Normal Mouse IgG antibody was used to perform IP followed by immunoblotting with anti-NSP5 antibody for B and anti-ACLY antibody for C and D, as shown in the IP panel. Inputs shown in the left panel, were probed with anti-ACLY antibody for A-D, anti-NSP5 antibody for A-B and anti-FLAG antibody for C and D. β-actin was used as an internal loading control for inputs.

**Fig S3:** **RV-NSP5-host protein interactome.** **(A)** STRING v12.0 was used to generate protein-protein interaction (PPI) network using 128 RV-NSP5 interacting host proteins present only in RV-SA11 infected MA104 cells in minimum of two biological replicates. K-means clustering was applied to identify group of proteins sharing common biological functions, indicated by red, yellow, green, cyan and blue (proteins belonging to distinct groups are mentioned in the table provided below). **(B)** PPI-network generated using 63 high-confidence proteins having at least one significantly unique peptide sequences from proteomics data. Proteins belonging to different groups after K-means clustering is represented by red, yellow, green, cyan and blue (proteins list for specific groups are provided in the table below). STRING v12.0 gene ontology (GO) enrichment analysis with FDR ≤0.001 was used to generate **(C)** Biological Process **(D)** Molecular Function **(E)** Cellular Component **(F)** KEGG pathways.

**Fig S4: RV-Wa infection induces ACLY ser455 phosphorylation; ACLY phosphorylation promotes NSP5-ACLY interaction, its knockdown impairs RV infection; and** **SB204990 inhibits infection at an early stage of RV life cycle.** lysates were prepared from MA104 cells at 0h, 3h, 6h, 9h and 12h from **(A)** mock infected and **(B)** RV-Wa infected cells at MOI 3. For infected cells, time of virus removal after 1 h of virus adsorption was considered as 0 hpi. Western blot analysis was done using anti-P-ACLY ser455, anti-ACLY, anti-VP6 and anti-β-actin antibodies. **(C)** SB204990 treatment confirms the phosphorylation dependent NSP5-ACLY interaction. **(D)** RV-SA11 infected MA104 cells were treated with DMSO or SB204990 (10μM) either 1 h prior to infection (pre-treatment) or after 1 h of virus adsorption at 0 hpi and 5 hpi (post-treatment) and viral titre was determined using plaque assay after 9 h. Unpaired t-test was used to determine the level of significance (***p* = 0.001, ****p* ≤ 0.001, ns denotes *p* value not significant). Each bar represents mean ±SD of three independent experiments. **(E)** MA104 cells were knockdown using siRNA-ACLY (50nM and 100nM) in RV-SA11 infected cells and VP6 expression was assessed. Blots were normalized against β-actin

**Fig S5: ACLY inhibitor, HCA shows anti-rotaviral activity both *in vitro* and *in vivo*. (A)** MA104 cells were treated with different concentrations of HCA (50μM, 100μM, 200μM, 400μM and 800μM) and after 72 h MTT assay was performed to calculate CC_50_. **(B**) Using the plaque assay, IC_50_ was calculated for HCA at different doses (25μM, 50μM, 100μM and 200μM) in RV-infected cells. **(C**) Western blot analysis was done to study dose-dependent effect of HCA on RV-VP6, P-ACLY ser455 and ACLY protein expression using anti-VP6, anti-P-ACLY ser455 and anti-ACLY antibodies. For internal loading control, anti-β-actin antibody was used**.** **(D)** Body weight measurement of uninfected mice treated orally with HCA at 5mg/kg/day, 10mg/kg/day and 15mg/kg/day to assess drug cytotoxicity. **(E)** On day 5, intestinal homogenates were prepared from RV-SA11 infected and HCA treated (10mg/kg/day and 15mg/kg/day) groups and protein expression of VP6 (infection marker) and β-actin (loading control) were analyzed by immunoblotting using anti-VP6 and anti- β-actin antibodies. **(F)** Body weight measurement following HCA (10mg/kg/day) treatment to assess the restoration of growth in RV-SA11 infected mice. **(G)** Stool samples from infected and HCA treated mice were collected and virus yield from stool samples was quantified. **(H)** H&E staining was used to study tissue morphology of small intestine from mock infected, RV-SA11 infected and HCA treated mice. Two-way ANOVA followed by Bonferroni's multiple comparisons test was used for A and C and unpaired Student’s t-test was performed for D to determine level of significance (****p* ≤ 0.001). Each bar represents mean body weight of mice ±SD of three independent experiments.

**Fig S6: ACLY knockdown reduces acetyl-CoA production and LDs formation. (A)** Acetyl-CoA assay was performed from empty vector or FLAG-NSP5 transfected cells treated either with si-NC (100nM) or siRNA-ACLY (100nM) (left panel). ACLY knockdown and FLAG-NSP5 transfection were confirmed by western blotting with anti-ACLY and anti-FLAG antibodies (right panel). MA104 cells either treated with **(B)** siRNA-ACLY (100nM) following RV-SA11 infection or with **(C)** SB204990 in mock infected control cells were fixed at 5hpi and processed for immunofluorescence microscopy. VMs were stained using anti-NSP5 antibody followed by counter-staining with rhodamine (anti-rabbit, red), and LDs were stained using BODIPY 493/503 (green). CTCF for both D and E were analyzed (right panel). Scale bar = 5 μm. Unpaired t-test was used to determine the level of significance (**p* ≤ 0.03, ***p* ≤ 0.006, ****p* ≤ 0.001). Each bar represents mean ±SD of three independent experiments.
